# Pharmacological modelling of dissociation and psychosis: an evaluation of the Clinician Administered Dissociative States Scale and Psychotomimetic States Inventory during nitrous oxide (‘laughing gas’)-induced anomalous states

**DOI:** 10.1007/s00213-022-06121-9

**Published:** 2022-03-26

**Authors:** Giulia G. Piazza, Georges Iskandar, Vanessa Hennessy, Hannah Zhao, Katie Walsh, Jeffrey McDonnell, Devin B. Terhune, Ravi K. Das, Sunjeev K. Kamboj

**Affiliations:** 1 Clinical Psychopharmacology Unit, Research Department of Clinical, Educational and Health Psychology, University College London, London, UK; 2 Department of Anaesthesia and Perioperative Medicine, University College London Hospital, London, UK; 3 Department of Psychology, Goldsmiths, University of London, London, UK

**Keywords:** Dissociation, Psychosis, Psychotomimesis, CADSS, Dissociative anaesthetic, Nitrous oxide, Ketamine, NMDA, Glutamate

## Abstract

**Rationale:**

A significant obstacle to an improved understanding of pathological dissociative and psychosis-like states is the lack of readily implemented pharmacological models of these experiences. Ketamine has dissociative and psychotomimetic effects but can be difficult to use outside of medical and clinical-research facilities. Alternatively, nitrous oxide (N_2_O) — like ketamine, a dissociative anaesthetic and NMDAR antagonist — has numerous properties that make it an attractive alternative for modelling dissociation and psychosis. However, development and testing of such pharmacological models relies on well-characterized measurement instruments.

**Objectives:**

To examine the factor structures of the Clinician Administered Dissociative States Scale (CADSS) and Psychotomimetic States Inventory (PSI) administered during N_2_O inhalation in healthy volunteers.

**Methods:**

Secondary analyses of data pooled from three previous N_2_O studies with healthy volunteers.

**Results:**

Effect sizes for N_2_O-induced dissociation and psychotomimesis were comparable to effects reported in experimental studies with sub-anaesthetic ketamine in healthy volunteers. Although, like ketamine, a three-factor representation of N_2_O-induced dissociation was confirmed, and a more parsimonious two-factor model might be more appropriate. Bayesian exploratory factor analysis suggested that N_2_O-induced psychosis-like symptoms were adequately represented by two negative and two positive symptom factors. Hierarchical cluster analysis indicated minimal item overlap between the CADSS and PSI.

**Conclusion:**

N_2_O and ketamine produce psychometrically similar dissociative states, although parallels in their psychosis-like effects remain to be determined. The CADSS and PSI tap largely non-overlapping experiences under N_2_O and we propose the use of both measures (or similar instruments) to comprehensively assess anomalous subjective states produced by dissociative NMDAR antagonists.

**Supplementary Information:**

The online version contains supplementary material available at 10.1007/s00213-022-06121-9.

## Introduction

A range of anomalous experiences or ‘non-ordinary waking states’ (Dittrich 1994) is reported across different psychopathologies. ‘Dissociation’ refers to a constellation of these states, ranging from mild (non-pathological) absorption states through to severe disorders of identity stability (Lynn et al. 2019; Lyssenko et al. 2018). While chronic or habitual dissociation is generally a feature of dissociative or severe personality disorders, transient dissociation is also experienced in various other psychological disorders (for example, panic disorder, PTSD, and somatoform disorders) and is increasingly recognized as a transdiagnostic symptom (Ellickson-Larew et al. 2020). Dissociation is generally considered to be a multifaceted construct and numerous descriptors have been applied to its subcomponents. The idea of distinct disordered detachment states, characterized by depersonalization and derealization on one hand, and compartmentalized experiences in the form, for example, of dissociative amnesia, on the other, has proven to be particularly influential (Brown 2006; Holmes et al. 2005).

Psychosis-like states also occur on a spectrum and, again, are common to a variety of psychological disorders. In fact, dissociation and psychotic symptoms are closely related and some of their elements may overlap (e.g. consciousness and ego disruption; Humpston et al. 2016; Moskowitz et al. 2009). Indeed, as noted by Giesbrecht et al. (2007), people with dissociative identity disorder have more first rank Schneiderian symptoms (hallucinations and thought-related delusions) than patients with schizophrenia. Overall, a robust relationship between dissociative symptoms and positive (and to a lesser extent, negative) psychotic symptoms has been observed in clinical and non-clinical groups (Longden et al. 2020), with dissociation being particularly linked to hallucinations, paranoia and delusions. Childhood trauma may be a common aetiological factor in both dissociation and psychosis, and some studies suggest a mediating role for dissociative experiences in the relationship between childhood trauma and psychotic episodes in adulthood (Perona-Garcelán et al. 2010, 2012; Sun et al. 2018; Varese et al. 2012). Understanding the biological basis and shared phenomenology of dissociation and psychotic states along with an ability to model these states experimentally may inform novel approaches to treatment and diagnosis across a range of psychiatric diagnoses. Pharmacological models in particular enable a temporary and circumscribed recapitulation of the biological dysfunction underlying these disorders.

Drugs that produce dissociative effects — particularly N-methyl-D-aspartate receptor (NMDAR)-modulating dissociative anaesthetics such as ketamine and phencyclidine — are also psychotomimetic (Mason et al. 2008). Ketamine acutely produces positive and negative psychosis-like symptoms and has been employed as a pharmacological model of psychosis (Krystal et al. 1994; Morgan et al. 2012; Corlett et al. 2007; Corlett et al. 2016), providing a strong empirical basis for a ‘glutamatergic dysfunction model’ of schizophrenia (Frohlich and Van Horn 2014). Nitrous oxide (N_2_O) is also a dissociative anaesthetic and, like ketamine, may derive some of its subjective and behavioural effects through non-competitive NMDA-receptor antagonism (Jevtović-Todorović et al. 1998; Mennerick et al. 1998), although opioid and other neurotransmitter systems are also likely to be affected by N_2_O and ketamine (Emmanouil and Quock 2007).

Existing self-report scales of psychosis-like and dissociative states were generally developed to assess naturally occurring symptoms in psychiatric disorders or at-risk mental states, although some have also been employed or adapted to assess drug-induced subjective changes (e.g. Mason et al. 2008). Despite the common use of these scales, few studies have examined their construct validity. In particular, it is unclear whether drug-induced anomalous experiences — as assessed using items from prototypical measures of dissociative and psychosis-like states — hang together in a similar way within the class of NMDAR antagonist-dissociative anaesthetics like ketamine and nitrous oxide, and more broadly, whether the structure of these experiences conforms to theoretical-clinical proposals. From an applied perspective, such measurement issues are important because subjective states may be predictive of the therapeutic response to NMDAR antagonists (e.g. in those treated for depression; Luckenbaugh et al. 2014) or their abuse potential (Kamboj et al. 2021).

The current study addressed several aims. Firstly, using data from three previous studies (two published, one unpublished) from our lab, we tested whether N_2_O reliably elicits dissociative and psychotomimetic effects. We present the pooled effects sizes from measures of these constructs — the Clinician Administered Dissociative States Scale (CADSS; Bremner et al. 1998) and Psychotomimetic States Inventory (PSI; Mason et al. 2008) alongside benchmarked values from studies of ketamine that used these same measures. Our intention was to determine the extent to which N_2_O and ketamine — each tested at subanaesthetic doses similar to those used in other experimental studies in healthy human volunteers (e.g. Beck et al. 2020) — produced similar increases in dissociation and psychosis-like symptoms. Secondly, we examined the latent factor structure of the CADSS and PSI. Specifically, by means of confirmatory factor analysis (CFA), we tested whether there was support for a pre-defined three-factor structure for the CADSS (Bremner et al. 1998) during N_2_O inhalation, while also preliminarily testing whether construct refinement was warranted by testing alternative (one- and two-factor) models. Due to the absence of a strong a priori basis for a specific dimensional structure of the PSI, exploratory factor analysis (EFA) was deemed appropriate for evaluating the latent structure of N_2_O-induced psychosis-like symptoms measured using this scale. Lastly, because of apparent content overlap, we examined the extent to which the two scales measured separate constructs using hierarchical cluster analysis.

## Method

All studies were approved by University College London Research Ethics Committee and conducted in compliance with the Declaration of Helsinki. All participants provided full written informed consent.

### Data and protocols

Data from our previously published studies (study 1: Das et al. 2018; study 2: Kamboj et al. 2021) were combined with unpublished data from our lab (study 3). The pooled sample size of participants receiving N_2_O was *n* = 160, which was the final sample size used in the CFA. The total number of participants receiving placebo in these studies was *n* = 69 (studies 2 and 3 only). Data in all cases were from healthy volunteers, although participants in Das et al. (2018) were purposively recruited for their hazardous drinking status (though none were alcohol dependent). In two of the studies (studies 1 and 3), N_2_O was administered in the context of prior memory reactivation, as part of a programme of research on retrieval-dependent memory modulation. The memory procedures were not expected to affect the measures of dissociation or psychotomimesis used in the current analyses.

Descriptive statistics are presented for pre- and peri-inhalation dissociation and psychotomimesis data, upon which mixed effects models were also conducted. However, CFA, EFA and hierarchical cluster analysis were only performed on peri-inhalation responses under N_2_O. Conducting such analyses on pre-N_2_O or peri-air CADSS and PSI data is unlikely to produce valid or meaningful findings given the preponderance of floor-level scores on all items under these conditions in healthy participants.

Across pre- and peri-inhalation data, 1.38% of item-level data were missing (11 single item responses in peri-inhalation CADSS data in study 1). Based on the estimator used in the CFA and EFA (see below), all available data from each pair of variables was used in estimating sample statistics. For the hierarchical cluster analysis, missing data were imputed with median values.

### Drugs

In all studies, participants inhaled Entonox (BOC, UK: 50% N_2_O premixed with 50% oxygen) for 30 min via an on-demand mouthpiece as outlined in Kamboj et al. (2021). In studies 2 and 3, participants randomized to a placebo condition inhaled medical air (BOC) for the same period. Assessments began after at least 5-min equilibration. Although the peri-inhalation CADSS and PSI were not administered at exactly the same time in the three studies, these assessments occurred within ~15 min of initiating inhalation. However, any variation in timing of assessment between studies is unlikely to have affected the results given the stability of blood gas levels after equilibration.

### Instruments

According to the original description of the CADSS (Bremner et al. 1998), its 19 self-report items can be divided into three subscales (number of items in brackets): amnesia (2), depersonalisation (5) and derealisation (12). Responses were recorded on a five-point scale: 0 = ‘not at all’ to 4 = ‘extremely’ scale. The CADSS is commonly used in experimental studies of ketamine (e.g. Curran and Monaghan 2001; Morgan et al. 2004; Zarate Jr et al. 2006; Aan Het Rot et al. 2010), and a recent paper describing a CFA of the CADSS during ketamine administration indicated that a three-factor model produced a good fit to the data (Niciu et al. 2018).

The PSI has 48 items, which were designed to assess distinct aspects of psychosis-like states: delusory thinking (8 items), perceptual distortions (10), cognitive disorganization (9), anhedonia (7), mania (6) and paranoia (8; Mason et al. 2008). Responses were recorded on a four-point scale: 0 = ‘not at all’ to 3 = ‘strongly’ scale. In previous pharmacological studies, the PSI was shown to be sensitive to the effects of cannabis and ketamine (Mason et al. 2008, 2009). Previous studies of the PSI described total and subscale scores and, to our knowledge, it has not yet been subjected to a factor analysis.

### Statistical analyses

#### Time-dependent drug effects and effect sizes

Descriptive statistics in the main text are reported as means ± standard deviations (SD). Linear mixed effect models were conducted using R (version 4.1.0) to assess fixed effects of time and drug (and their interaction) on CADSS and PSI scores using data from studies 2 and 3, which included a medical air control condition. Participant was a random-factor in these analyses. Significant effects were followed up with post hoc Bonferroni-corrected *t*-tests. The R package ‘lmerTest’ (Kuznetsova et al. 2015) was used to obtain p-values using Satterthwaite’s method for approximating degrees of freedom. Effect sizes were calculated as standardized mean differences (Cohen’s *d*) and associated 95% confidence intervals were obtained using the R package MBESS (Kelley 2020). Effect (sizes) of N_2_O on the CADSS and PSI were compared to benchmarked effects of ketamine from relevant publications (Dickerson et al. 2010; Mason et al. 2008). These studies respectively used a 0.23 mg/kg loading dose and infusion rate of 58 mcg/kg/min (Dickerson et al. 2010) and a 150 ng/ml target dose (Mason et al. 2008). Effect sizes (ES) and confidence intervals (95% CI) are based on peri-inhalation means (SDs) of N_2_O and placebo-medical air, and means (SDs) of post-infusion ketamine and placebo.

Where means and standard deviations were not reported numerically, these were obtained from the published figures using a plot digitiser (WebPlotDigitizer; Rohatgi 2021). Standard errors were converted to standard deviations.

### Factor analyses

The suitability of the data for factor analyses (Kaiser-Meyer-Olkin test, KMO; Bartlett's test of sphericity) was assessed with the R package ‘parameters’ (Lüdecke et al. 2020), although the actual factor analyses were performed on Mplus (version 8.6). Construct validity, based on the notion of a three component model of dissociation, was assessed using CFA of the CADSS during N_2_O inhalation (pooled *n* = 160). The ordinal nature of the indicator variables required the use of a weighted least squares mean and variance adjusted (WLSMV) estimator, which makes no distributional assumptions of the observed variables (Li 2016). This is particularly relevant for measuring anomalous experiences in healthy people, who on aggregate, will likely show a positively skewed pattern of responses in the on-drug condition when moderate (rather than high) doses are used. Examination of the distribution of item scores indicated that this was indeed true of most CADSS (and PSI, see below) items.

We tested the three-factor structure for the CADSS proposed by Bremner et al. (1998) using accepted cut-offs from standard fit indices (root mean square error of approximation, RMSEA; Comparative Fit Index, CFI; Tucker-Lewis Index, TLI; standardized root mean square residual, SRMR; Table 2 and Supplementary Tables 1 and 2). Following conventions (Hu and Bentler 1999), values of RMSE < .06, SRMR < .80 CFI > .95 and TLI > .95 were considered to reflect good fit. Due to a high correlation between amnesia and derealization subscales in the original three-factor structure, we examined a two-factor model with amnesia and derealization items loading onto one factor and depersonalization items loading onto another. We also examined the fit statistics of a two-factor model with all of the depersonalization and derealization items loading onto a single ‘detachment’ factor, along with the original two amnesia items loading onto a separate ‘compartmentalization’ factor. Finally, we evaluated a one-factor model comprising all items. Since these models were non-nested, model fit comparisons were made on the basis of approximated BIC values by re-running the models using maximum likelihood (ML) estimation. Note ML was used only for model comparison purposes. All other reported parameter values and fit indices were based on WLSMV estimation.

Owing to the inherent uncertainty regarding the optimal number of factors in the PSI, EFA was employed (with oblique Geomin rotation) to assess solutions with one to six factors using data from studies 2 and 3 (*n* = 100, peri-inhalation data). A Bayesian estimator was used, given the relatively small size of our sample for factor analysis. Bayesian EFA was shown to have better low sample size performance (Muthén and Asparouhov 2012a), as well as producing more accurate factor scores and correlations between factors in small sample sizes compared to maximum likelihood estimation (Muthén and Asparouhov 2012b). Given the lack of previous research upon which to base prior distributions, a diffuse (non-informative) prior was specified, and 50,000 MCMC iterations were conducted (Muthén 2010). The decision regarding the eventual number of factors to retain involved considering eigenvalues, total variance accounted for by retained factors, factor loadings and cross-loadings, inter-factor correlations and a concern to balance parsimony with a theoretically and conceptually sensible solution. During the process, if items had small loadings (< 0.3) or cross-loaded on > 1 factor (i.e. when the ratio of loadings on two factors was > 0.75), models were re-tested without those items.

### Cluster analysis

To assess the extent to which the CADSS and PSI measure similar or non-overlapping effects during N_2_O inhalation, we applied the exploratory (or unsupervised learning) technique of hierarchical cluster analysis, using the R package ‘dendextend’ (Galili 2015). We aimed to identify clusters of PSI and CADSS items based on their distance, maximizing the separation between clusters and minimizing intra-cluster distances (Denis 2020). Peri-inhalation items were standardized to vary between 0 and 1, as PSI and CADSS use different Likert scale ranges. A Gower’s general dissimilarity coefficient (Gower 1971) was used to compute a dissimilarity matrix, as this is a better measure of distance for categorical data in hierarchical cluster analysis (Everitt et al. 2011). Ward’s method (Ward 1963) was then used as the clustering procedure, which minimizes the total within-cluster error sum of squares (Everitt et al. 2011). We assessed the resulting clusters by means of a silhouette plot (Rousseeuw 1987), visualizing the average silhouette of our data (a measure of how well matched items are to their respective clusters).

## Results

### Participant characteristics

The average age of participants who received N_2_O was 25.7 (*SD* = 5.2), and medical air, 25.3 (*SD* = 6.6) years. Of *n* = 160 participants who received N_2_O (studies 1, 2 and 3), *n* = 77 were women and *n* = 83, men (the age and gender of participants in each of the three studies is summarized in Supplementary Table 3).

### Dissociative effects of N_2_O

The pooled data from studies 2 and 3 (Table 1) comparing medical air (*n* = 69) to N_2_O (*n* = 100) showed significant time × drug interactions on total CADSS scores (*F*[1167] = 47.37, *p* < .001, *η*^2^ = 0.22) (Supplementary Fig. 1a), derealisation (*F*[1167] = 49.41 *p* < .001, *η*^2^ = 0.23), amnesia (*F*[1167] = 20.85 *p* < .001, *η*^2^ = 0.11) and depersonalisation (*F*[1167] = 31.38 *p* < .001, *η*^2^ = 0.16). To allow comparison to ketamine, data (peri-infusion saline vs. peri-infusion ketamine) from Dickerson et al. (2010) are presented in Table 1. As can be seen in the table, the between-condition effect sizes for the total CADSS score for N_2_O (peri-medical air vs. peri-N_2_O: *d* = 1.01, 95% *CI* [0.68, 1.33]) and ketamine (peri-ketamine relative to peri-saline placebo: *d* = 1.14, 95% *CI* [0.57, 1.70]) were comparable. However, the pre-post infusion differences in CADSS scores reported in Dickerson et al. (2010) could be considered somewhat low for ketamine (~Δ7.68 points, see Table 1). Data from each of the three studies considered in this analysis is presented in Supplementary Table 4.

**Table 1:** Means (SDs) for the CADSS (total and subscale scores) and PSI (total based on 48 items and on the 40 item, four-factor model) from studies 2 and 3, each of which included a placebo (medical air) condition. Effect sizes are Cohen’s *d* with 95% *CIs*. For comparison, CADSS and PSI values are presented respectively from ∆Dickerson et al. (2010, *n* = 28; ketamine infusion protocol: 0.23 mg/kg loading dose and infusion rate of 58 mcg/kg/min) and Mason et al. (2008, *n* = 16 per drug group, target plasma ketamine level: 150 ng/ml). Effect sizes (ES) and confidence intervals (95% *CI*) are based on peri-inhalation means (SDs) of N_2_O and placebo-medical air, and means (SDs) of post-infusion ketamine and placebo

	Medical air		N2O		ES (95% *CI*)	Placebo		Ketamine		ES (95% *CI*)
	Pre-inhalation	Peri-inhalation	Pre-inhalation	Peri-inhalation		Pre-infusion	Post-infusion	Pre-infusion	Post-infusion	
CADSS										
Total	1.67 (2.96)	2.87 (5.19)	1.66 (3.05)	13.8 (13.4)	1.01 (0.68–1.33)	0.02 (1.29)^**∆**^	0.2 (1.33)^**∆**^	0.13 (0.99)^**∆**^	7.81 (9.71)^**∆**^	1.09 (0.53–1.66)
Amnesia	0.10 (0.39)	0.28 (0.80)	0.13 (0.42)	1.52 (2.12)	0.73 (0.4–1.04)					
Depersonalisation	0.35 (0.95)	0.84 (1.74)	0.54 (1.42)	3.88 (4.29)	0.87 (0.55–1.19)					
Derealisation	1.22 (2.19)	1.75 (2.92)	0.99 (1.80)	8.36 (8.13)	1.01 (0.68–1.33)					
PSI										
Total (48 items)	19.4 (12.5)	18.8 (15.5)	17.11 (12.34)	26.93 (20.41)	0.44 (0.13–0.75)	19.2 (10.7)	17.2 (10.3)	17.2 (10.3)	24.0 (10.9)	0.64 (−0.08–1.34)
Total (40 items)	16.5 (10.3)	16.20 (12.9)	14.80 (10.6)	23.60 (17.2)	0.48 (0.16–0.79)					

### Psychotomimetic effects of N_2_O

N_2_O also elicited psychotomimetic effects as measured by the PSI. Again, pooled data from studies 2 and 3 showed significant drug × time interactions on total PSI scores based on the original 48-item scale (*F*[1167] = 25.22, *p* < .001, *η*^2^ = 0.13) (Supplementary Fig. 1b). The interaction was virtually unchanged when the analysis was repeated with the new total score based on 40 items from the best fitting model from a factor analysis (see below; *F*[1167] = 27.00, *p* <.001, *η*^2^ = 0.14). The between-groups effect size for N_2_O-induced psychotomimesis compared to medical air (*d* = 0.44, 95% *CI* [0.13, 0.75]) was again comparable to the findings of Mason et al. (2008) with ketamine (*d* = 0.64, 95% *CI* [−0.08, 1.34]) as indicated by overlapping confidence intervals associated with effect size estimates (Table 1). It is worth noting however, that the absolute change in psychotomimesis and dissociation from pre- to peri-drug was somewhat larger in the N_2_O studies relative to ketamine, although there was also greater variability during N_2_O inhalation relative to ketamine infusion (Table 1).

### Confirmatory factor analysis of the CADSS

A three-factor model (Model 1: Bremner model; Table 2) based on pre-specified items loading onto latent factors for amnesia, depersonalization and derealization as originally proposed by Bremner et al. (1998) was found to have a good fit to the data based on various global goodness-of-fit indices (*CFI* = 0.96, *TLI* = 0.954, RMSEA = 0.069, 95% *CI* [0.055, 0.083]). However, the model χ^2^ test was highly significant (*χ*^2^[149] = 263.66, *p* < 0.001), and as such, modification indices (MIs) were examined to determine possible sources of model misspecification. However, none of the potential post-hoc data-driven modifications could be theoretically justified, and as such, none was made. The significant χ^2^ test result may therefore reflect the high sensitivity of this test, and its tendency to produce type I errors. Factor loading estimates for items (all > 0.5; Supplement Table 2 and Supplementary Fig. 4) suggest a reasonable degree of variance accounted for by their respective factors. Importantly, however, the three factors from this model were strongly correlated (depersonalization-derealization *r* = 0.884; depersonalization-amnesia *r* = 0.734; derealization-amnesia: *r* = 0.936), raising concerns that they might not represent separable constructs.

**Table 2:** Goodness of fit statistics for the main 3-factor and two competing 2-factor CFAs of the CADSS during N_2_O inhalation. In model 2 (depersonalization-derealization), F1 consisted of depersonalization and in F2, amnesia items were subsumed within the derealization factor. For model 3 (detachment-compartmentalization), F1 consisted of combined depersonalization and derealization items and F2, the two amnesia items. Model 4 consisted of one general ‘dissociation’ factor consisting of all CADSS items

	Model 1	Model 2	Model 3	Model 4
	3-factor (Bremner model)	2-factor (depersonalization- derealization model)	2-factor (detachment-comparmental-ization model)	1-factor model
Parameters	98	96	96	95
Chi-squared	263.662	269.828	303.250	307.539
Df	149	151	151	152
*p*-value	< 0.001	< 0.001	< 0.001	< 0.001
RMSEA (95% *CI*)	0.069 (0.055–0.083)	0.070 (0.056–0.083)	0.079 (0.066–0.092)	0.08 (0.067–0.093)
CFI	0.960	0.958	0.947	0.945
TLI	0.954	0.953	0.940	0.939
SRMR	0.058	0.059	0.065	0.065
BIC	8220.37	8217.42	8260.23	8265.87

*RMSEA*, Root Mean Square Index of Approximation; *CFI*, Comparative Fit Index; *TLI*, Tucker-Lewis Index; *SRMR*, standardized root mean square residual; *BIC*, Bayesian information criterion (approximate values obtained from Maximum Likelihood models)

We therefore tested a two-factor model that combined amnesia and derealization items into a single factor, with the other items retained in a second depersonalization factor (model 2: 2-factor depersonalization-derealization model). Overall, fit indices for model 2 were similar to model 1 (Table 2). Again, however, a high correlation between the two factors (*r* = 0.868) might suggests that they are conceptually difficult to distinguish. We also examined another two-factor model based on the detachment-compartmentalization conceptualization of dissociation (Brown 2006; Holmes et al. 2005) (Model 3: 2-factor detachment-compartmentalization model). This involved loading all of the depersonalization and derealization items onto a single detachment factor, and retaining the two dissociative amnesia items as indicators for a compartmentalization factor (*r* = 0.887). Finally, we tested a one-factor model (model 4), loading all items onto a single latent variable of ‘Dissociation’.

As can be seen from Table 2, model fit indices (other than the χ^2^ test) were generally in line with/close to recommended cut-offs for all models, although model 1 (Bremner model) and model 2 (depersonalization-derealization model) performed slightly better. Model 3 is conceptually suboptimal and likely to be psychometrically unstable, because of the large asymmetry in the number of indicators making up the two factors. In addition, while maximum likelihood-based approximate BIC values suggested comparable fits for models 1 and 2, model 3 clearly had a poorer fit than both of these models (∆BIC ~40), and model 4 was poorer still.

### Exploratory factor analysis of peri-inhalation PSI

The Kaiser-Meyer-Olkin (KMO) measure of sampling adequacy indicated our data was appropriate for factor analysis (*KMO* = 0.72) and Bartlett’s test of sphericity also suggested there was sufficient correlation for factor analysis (*χ*^2^[1128] = 3184.41, *p* < 0.001). Although the deflection on the screeplot of eigenvalues suggested a single factor solution (see Supplementary Fig. 2), such a model accounted for only 32% of common variance. On the other hand, a six-factor solution (e.g. as might be expected on the basis of PSI item design, which was intended to tap the six constructs of delusional thinking, paranoia, perceptual distortion, mania, cognitive disorganization and anhedonia) produced many cross-loadings, and one of the factors loaded on only one item. Sequential item removal did not adequately resolve these issues. Similar issues were encountered with a five-factor model. Of the remaining models, the most appropriate solution appeared to be a four-factor model formed from 40 of the original 48 PSI items, which accounted for 60% of variance. In an initial factor extraction, eight items (of the original 48) that had the most significant issues related to cross-loading or small factor loadings were removed. In the resulting 40-item model, average item loadings for all factors were > 0.6 and generally exceeded 0.5 (see Table 3). The 95% credible intervals for the loadings are presented in Supplementary Table 6. Moreover, items generally loaded cleanly on their respective factors (but see footnote of Table 3), and the values of Cronbach’s alpha for all four factors were acceptable (*α* ≥ 0.75; Table 3).

**Table 3:** Factors extracted from an EFA of 40 items from the PSI and Cronbach’s alpha values for the new factors (top), and correlation between factors (bottom). *Indicates one item that remained cross-loaded in the final model but was retained. The factor in which it was eventually retained was dictated by conceptual similarity with other items within that factor

	Original grouping	New factors			
		Negative I	Negative II	Positive I	Positive II
PSI 2	Cog Disorg	0.584	--	--	--
PSI 3	Mania	0.505	--	--	--
PSI 8	Cog Disorg	0.607	--	--	--
PSI 9	Anhedonia	0.580	--	--	--
PSI 10	Cog Disorg	0.794	--	--	--
PSI 13	Cog Disorg	0.747	--	--	--
*PSI 15	Anhedonia	0.465	--	--	--
PSI 16	Mania	0.835	--	--	--
PSI 28	Cog Disorg	0.717	--	--	--
PSI 30	Cog Disorg	0.617	--	--	--
PSI 34	Cog Disorg	0.862	--	--	--
PSI 41	Mania	0.621	--	--	--
PSI 46	Cog Disorg	0.642	--	--	--
PSI 47	Cog Disorg	0.569	--	--	--
PSI 1	Anhedonia	--	0.833	--	--
PSI 6	Anhedonia	--	0.732	--	--
PSI 18	Anhedonia	--	0.684	--	--
PSI 4	Delusion	--	--	0.326	--
PSI 7	Paranoia	--	--	0.571	--
PSI 12	Delusion	--	--	0.489	--
PSI 17	Paranoia	--	--	0.792	--
PSI 19	Delusion	--	--	0.504	--
PSI 20	Perc distortion	--	--	0.438	--
PSI 23	Paranoia	--	--	0.668	--
PSI 33	Paranoia	--	--	0.643	--
PSI 36	Perc distortion	--	--	0.750	--
PSI 39	Anhedonia	--	--	0.564	--
PSI 40	Delusion	--	--	0.928	--
PSI 42	Paranoia	--	--	0.728	--
PSI 43	Perc distortion	--	--	0.645	--
PSI 44	Perc distortion	--	--	0.918	--
PSI 45	Perc distortion	--	--	0.820	--
PSI 5	Perc distortion	--	--	--	0.479
PSI 22	Perc distortion	--	--	--	0.601
PSI 26	Delusion	--	--	--	0.721
PSI 27	Perc distortion	--	--	--	0.753
PSI 31	Delusion	--	--	--	0.725
PSI 32	Perc distortion	--	--	--	0.610
PSI 35	Delusion	--	--	--	0.606
PSI 48	Mania	--	--	--	0.568
Cronbach’s alpha		0.90	0.76	0.85	0.81
		Factor correlations			
	Negative I	1			
	Negative II	0.072	1		
	Positive I	0.482	0.097	1	
	Positive II	0.395	0.099	0.367	1

The four factors consisted of two negative symptom factors, which we provisionally refer to as ‘Negative I’, and ‘Negative II’ — and two positive symptom factors, labelled ‘Positive I’ and ‘Positive II’. Correlations between factors were all < 0.5, suggesting these were relatively distinct constructs. The Negative I factor consisted largely of items intended to tap the capacity to initiate and maintain organized thought (consisting of 7 out of 10 of the cognitive disorganization items from the originally proposed grouping of items, for example PSI-28 ‘…difficult to think clearly’; Mason et al. 2008). A number of items that were originally classified as mania-related (Mason et al. 2008) that generally tapped notions of cognitive overload (e.g. PSI-16: ‘…mind….full of ideas …can’t concentrate’) also formed part of this factor. Negative II consisted of three (reversed) social anhedonia items, based on the original anhedonia item grouping (Mason et al. 2008), for example PSI-1, ‘…enjoy mixing with people’.

Positive I consisted largely of paranoia, delusory thinking (such as, respectively, PSI-12 ‘…other people can read your mind’ and PSI-40, ‘…can read other people’s minds’) and thematically linked items of perceptual distortion (e.g. PSI-44, ‘…can sense an evil presence around you…’). Positive II also included delusory thinking items, such as PSI-26, ‘…thoughts so strong you can almost hear them’. In contrast with Positive I, items in the factor primarily related to the perceptual distortion of bodily sensations, (such as PSI-5 ‘…more sensitive to light…’). One of the items in Positive II belonged to the original mania grouping (Mason et al. 2008), although this item (PSI-48), which related to believing oneself to be a special person on an important mission, clearly also had a delusional flavour.

### Hierarchical cluster analysis of CADSS and PSI items

**Fig. 1:**
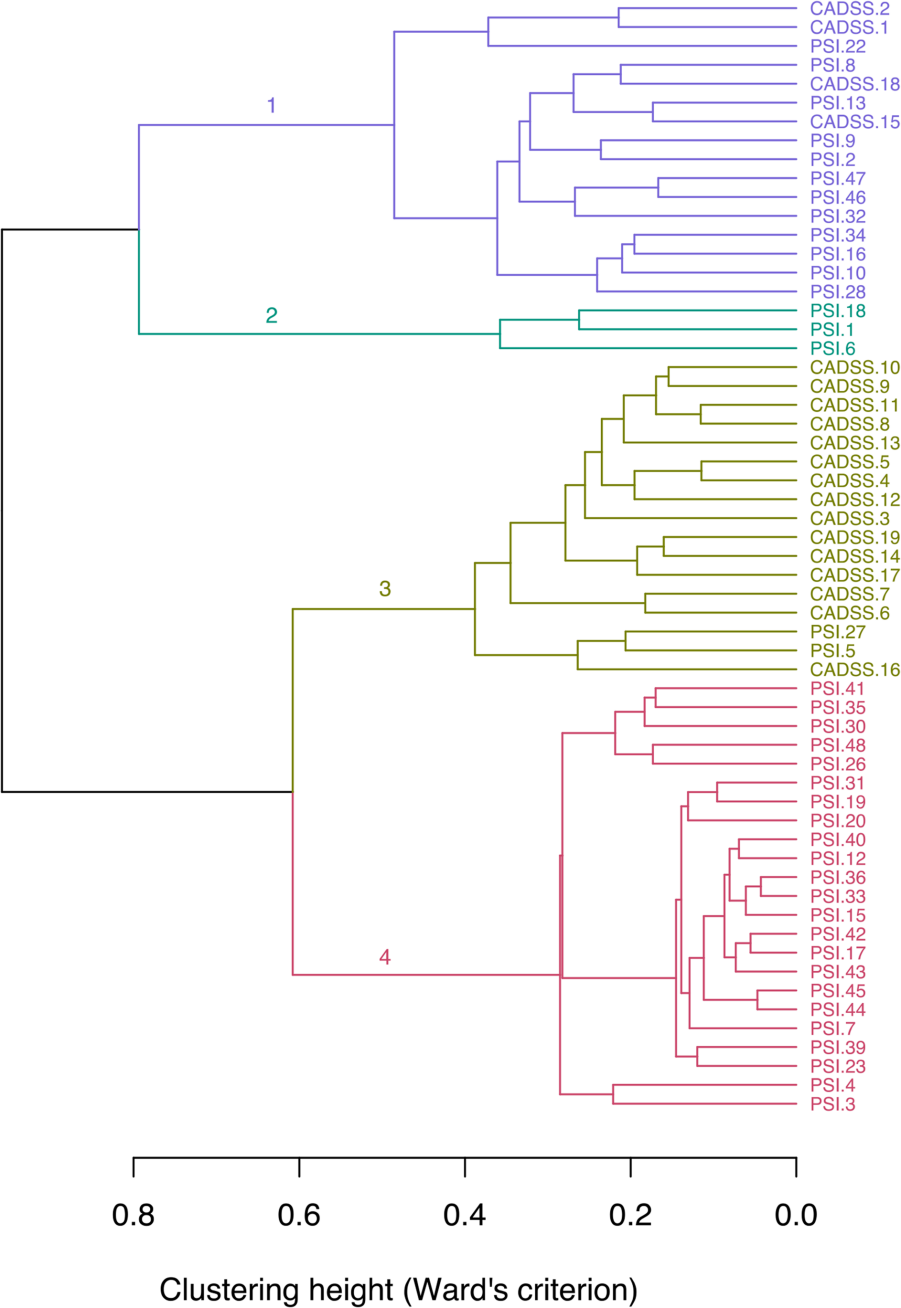
Dendrogram showing CADSS items and PSI items clusters based on a hierarchical cluster analysis, showing four different clusters of items (numbered)

The application of hierarchical cluster analysis to the CADSS and PSI items suggested that the items had limited overlap. A silhouette plot suggested four to be the optimal number of clusters (based on how well each item was matched to its cluster) (Supplementary Fig. 3). The first cluster (branch 1 in Fig. 1) consisted of PSI items from the Negative I factor and four CADSS items from the derealisation and amnesia subscales, which were thematically related to the content of items in Negative I, such as a lack of organised thought (e.g. CADSS-18 “*…*looking at the world through a fog”). A similar item from the Negative II factor (PSI-32, “…head, limbs or body have changed”) was grouped in this first cluster. The second cluster (branch 2) grouped together all items from factor Negative II, pertaining to social anhedonia. The third cluster (branch 3) involved all remaining 15 CADSS items and two PSI items from the perceptual distortion subscale/Positive II factor (PSI-27, PSI-5). The fourth cluster (branch 4) consisted positive psychosis-like experiences, including all items from the Positive I factor, most items from the Positive II factor and some items from Negative I (PSI-3, PSI-15, PSI-31, PSI-37, PSI-41) and Negative II (PSI-21). Overall, therefore, the findings are consistent with the EFA for the PSI but more importantly highlight the distinctiveness of dissociation and positive and negative psychosis-like symptoms induced by N_2_O.

## Discussion

This is the first study that we are aware of that has examined the latent factor structure of the CADSS under N_2_O-induced dissociation. It supplements the only other CFA of the CADSS, which was performed under conditions of ketamine-induced dissociation (Niciu et al. 2018). It is also the only study that we know of that has examined the factor structure of a self-report measure of drug-induced psychosis-like symptoms. Pooling data from separate studies from our lab, we showed clear, replicable increases in dissociation and psychosis-like states during N_2_O inhalation. Moreover, the between-group (N_2_O vs. medical air) effect sizes on both the CADSS and PSI were comparable to those reported with moderate-dose ketamine in healthy participants (Dickerson et al. 2010; Mason et al. 2008, respectively). Secondly, the CFA indicated that the psychometric structure of N_2_O-induced dissociation assessed using the CADSS conformed to a three-factor model, as originally proposed for dissociative disorders (Bremner et al. 1998), although a two-factor model appeared to be equally viable. Thirdly, while items of the PSI were originally designed to measure six distinct psychosis-like phenomena (delusory thinking, perceptual distortion, cognitive disorganisation, anhedonia, mania and paranoia; Mason et al. 2008), our EFA suggests that N_2_O-induced psychotomimesis has a simpler, four-factor structure (based on 40 of the originally devised 48 item questionnaire). This consisted of two negative and two positive symptom factors. Finally, although phenomenological parallels and co-occurrence of psychosis-like and dissociative symptoms have been noted, cluster analyses of the PSI and CADSS indicated that these are largely non-overlapping constructs.

Despite its prevalence as a transdiagnostic symptom across a range of psychopathologies (Ellickson-Larew et al. 2020; Lyssenko et al. 2018), there are currently no widely accepted pharmacological or behavioural models of dissociation. Although dissociative states are an observed effect of ketamine (e.g. Morgan et al. 2010), researchers have not generally used ketamine to specifically provoke dissociation, instead generally viewing dissociation as an off-target effect in clinical trials for depression (e.g. Niciu et al. 2018; Włodarczyk et al. 2021). However, there is some evidence that dissociation is an important predictor of antidepressant response to ketamine (Niciu et al. 2018; Mathai et al. 2020). Some behavioural methods (e.g. extended mirror gazing) also produce dissociative symptoms (Nisticò et al. 2020), although these have significant limitations. For example, it is likely that behaviourally induced states are relatively fragile and easily disrupted by the measurement procedure (a likely general problem with behavioural techniques for inducing anomalous states, perhaps with the exception of hypnosis). As with the CO_2_ model of anxiety (Bailey et al. 2011), as an inhalable gas, the N_2_O model of dissociation represents a relatively easily implemented experimental method for dissociative symptom provocation. It is less invasive, and symptoms reverse more quickly than with ketamine; it has fewer regulatory obstacles associated with its use (at least in the UK, at this time of writing), and unlike behavioural methods, it does not require retrospective assessment of symptoms. Finally, because there is virtually no metabolism of N_2_O prior to respiratory excretion, dissociative effects can be attributed to N_2_O alone. This might not be the case for ketamine, which produces a number of psychoactive metabolites that may have distinct dissociative effects over the course of infusion. These considerations suggest that N_2_O is a viable pharmacological model of dissociation that warrants further investigation.

The validation of a pharmacological model of dissociation requires the use of validated measures that adequately capture its fundamental phenomenology. While we did not perform a complete validation of the CADSS, construct validity was tested using CFA. The three-factor model, as originally proposed (but not tested) by Bremner et al. (1998), produced a good fit to the data. On the other hand, the strong correlation between factors raises concerns about the appropriateness of a three-factor model, at least as applied to N_2_O-induced dissociation. Surprisingly, there are very few factor analytic studies of the CADSS, and only one CFA that we could identify (Niciu et al. 2018). That study examined ketamine-induced dissociation and also suggested that the originally proposed three-component model of dissociation provided good fit. However, Niciu et al. (2018) did not test any competing models, and it is not clear if, like N_2_O-induced dissociation in the present study, a two-factor depersonalization-derealization model (in which derealization and amnesia items load on a single factor) is equally valid for ketamine-induced dissociation. Indeed, it is likely that collapsing the amnesia factor into derealization would produce more stable and generalizable findings, given that amnesia is only formed of two indicators (DiStefano and Hess 2005). In fact, considering the content of the amnesia items (item 14: ‘Do things happen that you later cannot account for?’; item 15: ‘Do you space out, or in some other way lose track of what is going on?’), it could be argued that they are closer to the forms of absorption or feelings of being ‘spaced out’ that are commonly associated with detachment (Butler et al. 2019) and as such, belong in one of the two detachment factors rather than a separate compartmentalization-related factor. Furthermore, although the CADSS compartmentalization items have occasionally shown some discriminant validity (Nisticò et al. 2020), this feature of dissociation is under-represented on this scale and additional items or alternative measures are required to more adequately assess the compartmentalization dimension of drug-induced dissociation. Indeed, while our findings suggest that N_2_O-induced dissociation might resemble the structure of such experiences in dissociative disorders, they do not speak to whether the CADSS captures all or even the most important aspects of N_2_O-dissociation. For example, the CADSS does not include depersonalization items relating to disconnection from emotions, although this seems like a relevant assessment domain for dissociative anaesthetics, particularly in the context of research on the antidepressant effects of N_2_O and ketamine (Nagele et al. 2015; Nagele et al. 2018; Singh et al. 2016; Zarate Jr et al. 2006). Indeed, systematic descriptions of dissociation-related experiences suggest that a variety of different ‘types’ of dissociation (e.g. unreality, automaticity, disconnection) affecting a number of domains (e.g. the body, affect, cognition, perception, identity, see Butler et al. 2019; Černis et al. 2021) could potentially be important in N_2_O-induced dissociation. Similar considerations are relevant for measuring dissociation in studies of ketamine and other drugs with dissociative effects (e.g. cannabis; van Heugten-Van der Kloet et al. 2015). Future studies might therefore consider using more comprehensive measures of dissociative phenomena, suitably adapted to measuring state changes. An additional consideration is the individual differences factors that predispose individuals to dissociative states under the influence of N_2_O, with implications for therapeutic applications of this agent. Preliminary work suggests that dissociative tendencies predict dissociative responses to ketamine (as indexed by the CADSS) (Mello et al. 2021), but corresponding individual differences research is required with N_2_O-induced dissociative states.

Studies of pharmacologically induced psychosis-like symptoms have been dominated by experiments with ketamine (see “Introduction”). Here, we show that nitrous oxide produces a similar degree of (self-reported) psychotomimesis to moderate-dose ketamine. There are relatively few studies that use self-report measures of psychosis-like drug effects, with most studies of ketamine instead employing clinician-administered scales. While these scales seem appropriate for more severe symptoms, we contend that for relatively mild drug-induced symptoms, self-report scales like the PSI may be more appropriate for capturing multiple, psychosis-like subjective states, which rely on introspection (although of course, this also approach has limitations). The original PSI items were intended to capture six different psychosis-like domains, although our EFA supports a simpler, four-factor structure during N_2_O inhalation. Whether such a four-factor model of drug-induced psychotomimesis is specific to N_2_O or generalizes to other dissociative-NMDAR antagonists like ketamine is unclear.

A hierarchical cluster analysis provided further support for distinct psychotomimetic states, as well as their distinctness from dissociative states. However, it is of note that this analysis found that the CADSS cluster (third cluster) was more similar to the PSI cluster primarily comprising positive symptoms. This conceptually aligns with meta-analytic research showing dissociative states are more strongly associated with positive than negative symptoms in clinical and non-clinical samples (Longden et al. 2020). At this stage, we can contend that N_2_O, like ketamine, produces both positive and negative psychosis-like symptoms and that the overall effect size, as measured by total PSI scores, is similar to that seen with ketamine (based on overlapping confidence intervals for the two drugs). It therefore seems worthwhile to further examine whether, like ketamine, N_2_O produces disruptions in for example, associative learning, working memory and attentional control that resemble the initial phases of delusion formation in psychosis (Corlett et al. 2007).

A number of limitations of the current study must be acknowledged. Firstly, the study involved post hoc analysis with no pre-registration of the analysis plan. Secondly, the sample size in our factor analyses might be considered rather small, especially for the PSI. On the other hand, our use of Bayesian EFA in our analysis of the PSI obviated some of the issues associated classical approaches to factor analyses with small samples. Another limitation is that the CFA was only conducted on cross-sectional data; factor invariance was not assessed.

In conclusion, we found that N_2_O reliably elicited dissociative and psychotomimetic effects that were similar in magnitude to those reported with ketamine. This provides preliminary validation for the use of N_2_O as a pharmacological model of dissociation and motivates further research on N_2_O-induced psychotic states in healthy volunteers. Although a three-factor structure for the CADSS produced a good fit to our data (based on CFI, TLI and RMSEA values), a consideration of the distribution of items across the three factors, as well as item wording, might suggest that items that assess ‘dissociative amnesia’ should be subsumed within the derealization factor. Indeed, a two factor model yielded comparably good fit indices to the three-factor model. Future pre-registered analyses examining competing models might help to resolve this issue. Similar analyses applied to ketamine-induced dissociation will allow us to draw firmer conclusions about the similarity of these two drugs in terms of the nature of the dissociative symptoms they produce. We suggest that while the psychometric behaviour of the CADSS should continue to be investigated, additional, more comprehensive measures of dissociation may be required to more fully assess dissociative (particularly compartmentalization-related) phenomena produced by dissociative anaesthetics. Additional work is required to determine whether an N_2_O-model would be a valid alternative model of NMDAR dysfunction in psychosis. In addition to further construct validation using the PSI, neuropsychological and electrophysiological evidence is required to fully evaluate an N_2_O-model of psychosis. Such a model would offer multiple advantages over the ketamine model in terms of safety, convenience, and rapid reversibility of side effects. Once further validated, use of self-report scales for assessment of drug-induced positive and negative psychosis symptoms — like the PSI — would also offer advantages in terms of convenience and efficiency over clinician-administered scales.

## Supplementary Information

Below is the link to the electronic supplementary material.

Supplementary file1 (DOCX 522 kb)
