# Supplementary material for: Pharmacological modelling of dissociation and psychosis: an evaluation of the Clinician Administered Dissociative States Scale and Psychotomimetic States Inventory during nitrous oxide (‘laughing gas’)-induced anomalous states
Source: Psychopharmacology (Berl). 2022 Mar 26;239(7):2317–29. doi: 10.1007/s00213-022-06121-9 (PMC9205822; doi:10.1007/s00213-022-06121-9)
Supplement: Supplementary file 1 — Supplementary file1 (DOCX 522 kb) [file 213_2022_6121_MOESM1_ESM.docx]

**Pharmacological modelling of dissociation and psychosis: An evaluation of the Clinician Administered Dissociative States Scale and Psychotomimetic States Inventory during nitrous oxide (‘laughing gas’)-induced anomalous states.**

**Supplementary Information**

Giulia G Piazza^1^, Georges Iskandar^1,2^, Vanessa Hennessy^1^, Hannah Zhao^1^, Katie Walsh^1^, Jeffrey McDonnell^1^, Devin B. Terhune^3^, Ravi K Das^1^, Sunjeev K Kamboj^1*^

^*^Corresponding author: sunjeev.kamboj@ucl.ac.uk

^1^Clinical Psychopharmacology Unit, Research Department of Clinical, Educational and Health Psychology, University College London

^2^Department of Anaesthesia and Perioperative Medicine, University College London Hospital, UK

^3^Department of Psychology, Goldsmiths, University of London

**Supplementary Figure 1a and 1b:** Raincloud plots (Allen et al, 2018; Allen et al, 2021) showing raw data, probability density and boxplots with median values of (a) total scores on the CADSS and (b) total scores on the PSI for placebo (medical air) and N_2_O conditions pre- and peri-inhalation.

a.

b.

**Supplementary Figure 2:** Scree plot of eigenvalues used to establish the optimal factor structure of the PSI with EFA^⁋^.

******^⁋^Although the plot might suggest a single factor, as can be seen, there were a number of factors with eigenvalues >2. In addition, other factors (see main paper) were taken into consideration when determining the number of factors to retain. Parallel analysis was not conducted, as not advised when dealing with categorical data.

**Supplementary Figure 3:** Plot showing the number of possible clusters in hierarchical cluster analysis against average silhouette width to determine the optimal number of clusters (red circle, 4 clusters).

**Supplementary Table 1**: Supplementary table to table 1 in the main paper. Means and SDs for the PSI subscales proposed by Mason et al (2008; pooled data from Studies 2 and 3; total N_2_O: n=100; total medical air: n=69) and for the four factors suggested by EFA. In addition to the Drug x Time interaction on total PSI reported in the main paper, similar interactions were found using the subscales originally proposed by Mason et al (2008) on the PSI: Delusory thinking [F(1,167 = 9.034, p =0.003, η^2^= 0.05], perceptual distortion [F(1,167) = 42.47, p <.0001, η^2^= 0.20], cognitive disorganisation [F(1,167) = 21.40, p <.0001, η^2^= 0.11], mania [F(1,167) = 13.11, p = 0.0003, η^2^= 0.07] and paranoia [F(1,167) = 6.06, p = 0.01, η^2^= 0.04]. No such interaction was found on anhedonia [F(1,167) = 1.01, p = 0.32]. Similar interactions were also found for three of the proposed four factors derived from EFA: Negative I [F(1,167) = 18.26, p <.001, η^2^= 0.14], Positive I [F(1,167) = 5.93, p < .05, ].η^2^= 0.03] and Positive II [F(1,167) =47.05, p < .001, η^2^= 0.22], but not for factor Negative II [F(1, 167) = 0.02, p = 0.88]

|  |  | |  | |  | |  | |
| --- | --- | --- | --- | --- | --- | --- | --- | --- |
|  | **Medical air** | | **N2O** | | **Placebo** | | **Ketamine** | |
|  | Pre-inhalation | Peri-inhalation | Pre-inhalation | Peri-inhalation | Pre-infusion | Post-infusion | Pre-infusion | Post-infusion |
| **Original six subscales** |  |  |  |  |  |  |  |  |
| Delusory Thinking | 2.10 (2.58) | 1.63 (1.43) | 2.04 (2.53) | 2.81 (3.71) | 2.3 (2.4) | 0.9 (1.3) | 1.2 (1.7) | 2.1 (2.2) |
| Perceptual Distortion | 0.8 (1.43) | 1.38 (2.9) | 0.96 (2.07) | 4.83 (4.24) | 1.0 (1.9) | 1.3 (2.0) | 1.3 (2.4) | 3.9 (3.1) |
| Cognitive Disorganisation | 4.74 (4.13) | 5.1 (4.57) | 3.77 (4.41) | 7.58 (6.82) | 6.5 (4.7) | 5.5 (4.8) | 5.1 (4.5) | 9.4 (5.1) |
| Anhedonia | 7.13 (2.39) | 6.42 (3.02) | 6.33 (2.67) | 6.03 (3.43) | 4.7 (2.5) | 5.1 (2.7) | 4.1 (2.2) | 5.6 (3.9) |
| Mania | 3.1 (2.67) | 2.8 (2.51) | 2.77 (2.85) | 3.68 (3.24) | 3.3 (1.5) | 3.6 (1.6) | 3.6 (2.9) | 3.4 (1.7) |
| Paranoia | 1.58 (2.39) | 1.45 (2.93) | 1.24 (1.92) | 2.00 (3.13) | 1.44 (1.5) | 0.6 (1.3) | 0.94 (0.8) | 0.56 (0.7) |
| **Four EFA factors** |  |  |  |  |  |  |  |  |
| Negative I | 6.96 (5.83) | 7.25 (5.87) | 5.81 (6.47) | 9.97 (8.65) |  |  |  |  |
| Negative II | 5.30 (2.07) | 4.68 (2.64) | 5.07 (2.49) | 4.4 (2.78) |  |  |  |  |
| Positive I | 2.83 (3.66) | 2.54 (5.13) | 2.33 (2.84) | 2.54 (5.13) |  |  |  |  |
| Positive II | 1.42 (2.27) | 1.75 (2.94) | 1.58 (2.27) | 5.69 (5.32) |  |  |  |  |

**Supplementary Table 2:** Standardised coefficients from the confirmatory factor analysis of the 3-factor structure of the CADSS in N_2_O. The data is presented in a similar way to the supplementary material of Niciu et al (2018) to allow direct comparison.

|  |  | **Estimate** | **SE** |
| --- | --- | --- | --- |
| **Derealization** |  |  |  |
| Things moving in slow motion | CADSS.1 | 0.595 | 0.058 |
| Unreal/ as if in a Dream | CADSS.2 | 0.785 | 0.040 |
| People seem motionless etc. | CADSS.8 | 0.772 | 0.051 |
| Objects look different | CADSS.9 | 0.747 | 0.044 |
| Colours diminished | CADSS.10 | 0.566 | 0.071 |
| See things as if in a Tunnel | CADSS.11 | 0.750 | 0.048 |
| Questionnaire taking longer | CADSS.12 | 0.694 | 0.051 |
| Things happening very quick | CADSS.13 | 0.636 | 0.060 |
| Sounds disappeared/become stronger | CADSS.16 | 0.696 | 0.047 |
| Things very real/special clarity | CADSS.17 | 0.587 | 0.065 |
| Looking at world through a fog | CADSS.18 | 0.663 | 0.049 |
| Colours seem brighter | CADSS.19 | 0.682 | 0.053 |
|  |  |  |  |
| **Depersonalization** |  |  |  |
| Separate from things | CADSS.3 | 0.791 | 0.043 |
| Looking at things outside of your body | CADSS.4 | 0.886 | 0.029 |
| Watching as an observer/spectator | CADSS.5 | 0.838 | 0.034 |
| Disconnected from own body | CADSS.6 | 0.811 | 0.033 |
| Body feels changed | CADSS.7 | 0.761 | 0.045 |
|  |  |  |  |
| **Amnesia** |  |  |  |
| Things happened, can’t account for | CADSS.14 | 0.820 | 0.055 |
| Space out, lost track | CADSS.15 | 0.808 | 0.041 |

**Supplementary Figure 4**: Path diagram of standardised coefficients and standard errors (in parentheses) from the CFA of the 3-factor structure of the CADSS during N_2_O inhalation. See Supplementary Table 2 for item content.

**Supplementary Table 4:** Descriptive statistics of the three studies described in the main paper. Means (SDs) are reported for age and years in education, while the ratio Male:Female and Caucasian:Other are reported for Gender and Ethnicity respectively.

x = Age in Study 2 is based on n = 64 (as it was not recorded for n = 6 medical air and n = 8 N2O participants).

|  | **Study 1 (Das et al., 2018)** | | **Study 2 (Kamboj et al., 2021)** | | **Study 3** | |
| --- | --- | --- | --- | --- | --- | --- |
|  | Medical air | N_2_O (n=60) | Medical air (n = 40) | N_2_O (n= 40) | Medical air (n=29) | N_2_O (n=60) |
| **Age** | - | 26.27 (8.4) | 26.09 (4.92)^x^ | 23.88 (3.66)^x^ | 25.28 (5.66) | 25.15(5.77) |
| **Gender (M:F)** | - | 37:23 | 19:21 | 17:23 | 14:15 | 29:31 |
| **Ethnicity (Caucasian:Other)** | - | 34:26 | 19:21 | 23:17 | 33:27 | 18:11 |

**Supplementary Table 5**: Descriptive statistics, mean (SD) for the pre- and peri timepoints for the CADSS and PSI for each of the three studies described in the main paper.

|  |  |  |  |  |  |  |  |  |  |  |  |  |  |  |  |  |  |  |
| --- | --- | --- | --- | --- | --- | --- | --- | --- | --- | --- | --- | --- | --- | --- | --- | --- | --- | --- |
|  | **Study 1 (Das et al., 2018)** | | | | **Study 2 (Kamboj et al., 2021)** | | | | | | | | **Study 3** | | | | | |
|  | Placebo | | N_2_O | | | Placebo | | | N_2_O | | | Placebo | | | N_2_O | | | |
|  | Pre | Peri | Pre | Peri | | Pre | Peri | | Pre | Peri | | Pre | | Peri | Pre | Peri | |  |
| **CADSS** |  |  |  |  | |  | |  |  | |  |  | |  |  | |  |  |
| Total | - | - | 3.64 (6.16) | 19.03 (13.35) | | 1.72 (2.60) | | 2.85 (6.08) | 1.52 (3.28) | | 14.2 (13.2) | 1.59 (3.45) | | 2.90 (3.74) | 1.75 (2.90) | | 13.5 (13.7) |  |
| Amnesia | - | - | 0.73 (1.28) | 2.13 (2.19) | | 0.15 (0.48) | | 0.23 (0.86) | 0.13 (0.52) | | 1.48 (1.92) | 0.04 (0.19) | | 0.35 (0.72) | 0.13 (0.34) | | 1.55 (2.26) |  |
| Depersonalisation | - | - | 0.81 (2.19) | 4.78 (4.23) | | 0.33 (0.86) | | 0.78 (1.97) | 0.5 (1.74) | | 4.05 (4.21) | 0.38 (1.08) | | 0.93 (1.39) | 0.57 (1.17) | | 3.77 (4.37) |  |
| Derealisation | - | - | 2.10 (3.45) | 12.12 (8.35) | | 1.25 (1.96) | | 1.85 (3.39) | 0.9 (1.68) | | 8.62 (8.44) | 1.17 (2.51) | | 1.62 (2.16) | 1.05 (1.89) | | 8.18 (7.99) |  |
| **PSI (original subscales)** |  |  |  |  | |  | |  |  | |  |  | |  |  | |  |  |
| Total | - | - | - | - | | 18.6 (14.4) | | 17.7 (18.9) | 13 (11.2) | | 22.6 (17.7) | 20.6 (9.24) | | 20.2 (9.12) | 19.8 (12.4) | | 29.8 (21.7) |  |
| Anhedonia | - | - | - | - | | 6.98 (2.63) | | 5.82 (3.44) | 5.68 (2.87) | | 5.08 (3.03) | 7.34 (2.04) | | 7.24 (2.12) | 6.77 (2.46) | | 6.67 (3.56) |  |
| Delusory think | - | - | - | - | | 2.1 (2.93) | | 1.62 (3.20) | 1.55 (2.58) | | 2.33 (2.80) | 2.10 (2.06) | | 1.66 (2.42) | 2.37 (2.47) | | 3.13 (4.20) |  |
| Perceptual Distort | - | - | - | - | | 0.825 (1.57) | | 1.42 (3.32) | 0.55 (1.13) | | 4.28 (3.92) | 0.76 (1.24) | | 1.31 (2.24) | 1.23 (2.49) | | 5.2 (4.44) |  |
| Cognitive Disorg | - | - | - | - | | 4.12 (4.15) | | 4.53 (5.09) | 2.48 (3.68) | | 6.48 (6.78) | 5.59 (4.01) | | 5.83 (3.69) | 4.63 (4.67) | | 8.32 (6.81) |  |
| Mania | - | - | - | - | | 3.05 (3.16) | | 2.65 (2.89) | 1.85 (2.27) | | 2.95 (2.73) | 3.10 (1.84) | | 3.07 (1.91) | 3.38 (3.04) | | 4.17 (3.48) |  |
| Paranoia | - | - | - | - | | 1.48 (2.40) | | 1.68 (3.46) | 0.9 (1.60) | | 1.5 (2.35) | 1.72 (2.42) | | 1.14 (2.01) | 1.47 (2.09) | | 2.33 (3.54) |  |
| **PSI (four EFA factors)** |  |  |  |  | |  | |  |  | |  |  | |  |  | |  |  |
| Total (40 items) | - | - | - | - | | 16.0 (12.1) | | 15.4 (15.6) | 11.50 (9.83) | | 20.20 (14.8) | 17.1 (7.52) | | 17.3 (7.91) | 17.00 (10.7) | | 25.8 (18.4) |  |
| Negative I | - | - | - | - | | 6.35 (6.02) | | 6.52 (6.49) | 3.85 (5.21) | | 8.35 (7.83) | 7.79 (5.56) | | 8.24 (4.81) | 7.12 (6.93) | | 11.00 (9.05) |  |
| Negative II | - | - | - | - | | 5.28 (2.21) | | 4.35 (2.81) | 4.62 (2.57) | | 4.03 (2.91) | 5.34 (1.9) | | 5.14 (2.36) | 5.37 (2.41) | | 4.65 (2.69) |  |
| Positive I | - | - | - | - | | 2.81 (3.99) | | 2.75 (6.04) | 1.8 (2.71) | | 2.75 (3.90) | 2.83 (3.22) | | 2.24 (3.59) | 2.68 (2.90) | | 4.03 (5.73) |  |
| Positive II | - | - | - | - | | 1.60 (2.6) | | 1.78 (3.13) | 1.20 (2.26) | | 5.05 (4.96) | 1.17 (1.71) | | 1.72 (2.72) | 1.83 (2.27) | | 6.12 (5.54) |  |

|  | **95% Credible Intervals for New Factors** | | | | | | | |
| --- | --- | --- | --- | --- | --- | --- | --- | --- |
|  | **Negative I** | | **Negative II** | | **Positive I** | | **Positive II** | |
| **PSI2** | 0.179 | 0.857 |  |  |  |  |  |  |
| **PSI3** | 0.114 | 0.798 |  |  |  |  |  |  |
| **PSI8** | 0.206 | 0.878 |  |  |  |  |  |  |
| **PSI9** | 0.165 | 0.855 |  |  |  |  |  |  |
| **PSI10** | 0.464 | 0.993 |  |  |  |  |  |  |
| **PSI13** | 0.352 | 0.964 |  |  |  |  |  |  |
| **PSI15** | 0.047 | 0.869 |  |  |  |  |  |  |
| **PSI16** | 0.517 | 1.028 |  |  |  |  |  |  |
| **PSI28** | 0.32 | 1.007 |  |  |  |  |  |  |
| **PSI30** | 0.2 | 0.914 |  |  |  |  |  |  |
| **PSI41** | 0.202 | 0.899 |  |  |  |  |  |  |
| **PSI46** | 0.259 | 0.895 |  |  |  |  |  |  |
| **PSI47** | 0.143 | 0.859 |  |  |  |  |  |  |
| **PSI1** |  |  | 0.561 | 0.96 |  |  |  |  |
| **PSI6** |  |  | 0.452 | 0.901 |  |  |  |  |
| **PSI18** |  |  | 0.416 | 0.871 |  |  |  |  |
| **PSI34** |  |  | -0.026 | 0.384 |  |  |  |  |
| **PSI4** |  |  |  |  | -0.15 | 0.735 |  |  |
| **PSI7** |  |  |  |  | 0.101 | 0.878 |  |  |
| **PSI12** |  |  |  |  | 0.031 | 0.832 |  |  |
| **PSI17** |  |  |  |  | 0.346 | 1.012 |  |  |
| **PSI19** |  |  |  |  | 0.004 | 0.867 |  |  |
| **PSI20** |  |  |  |  | 0.005 | 0.793 |  |  |
| **PSI23** |  |  |  |  | 0.253 | 0.915 |  |  |
| **PSI33** |  |  |  |  | -0.047 | 1.019 |  |  |
| **PSI36** |  |  |  |  | 0.264 | 0.994 |  |  |
| **PSI39** |  |  |  |  | 0.1 | 0.882 |  |  |
| **PSI40** |  |  |  |  | 0.574 | 1.1 |  |  |
| **PSI42** |  |  |  |  | 0.263 | 0.99 |  |  |
| **PSI43** |  |  |  |  | 0.228 | 0.926 |  |  |
| **PSI44** |  |  |  |  | 0.525 | 1.096 |  |  |
| **PSI45** |  |  |  |  | 0.377 | 1.028 |  |  |
| **PSI5** |  |  |  |  |  |  | 0.045 | 0.821 |
| **PSI22** |  |  |  |  |  |  | 0.2 | 0.906 |
| **PSI26** |  |  |  |  |  |  | 0.352 | 0.994 |
| **PSI27** |  |  |  |  |  |  | 0.324 | 1.011 |
| **PSI31** |  |  |  |  |  |  | 0.363 | 1.008 |
| **PSI32** |  |  |  |  |  |  | 0.202 | 0.947 |
| **PSI35** |  |  |  |  |  |  | 0.225 | 0.915 |
| **PSI48** |  |  |  |  |  |  | 0.174 | 0.879 |
|  | **95% Credible Intervals for Factor Correlations** | | | | | | | |
| **Negative I** | 1 | 1 |  |  |  |  |  |  |
| **Negative II** | -0.193 | 0.331 | 1 | 1 |  |  |  |  |
| **Positive I** | 0.179 | 0.695 | -0.173 | 0.353 | 1 | 1 |  |  |
| **Positive II** | 0.06 | 0.628 | -0.153 | 0.346 | 0.026 | 0.638 | 1 | 1 |

**Supplementary Table 6**: 95% Credible Intervals for new factors extracted from a Bayesian EFA of the PSI
